# Solid Organ Rejection following SARS-CoV-2 Vaccination or COVID-19 Infection: A Systematic Review and Meta-Analysis

**DOI:** 10.3390/vaccines10081289

**Published:** 2022-08-10

**Authors:** Saad Alhumaid, Ali A. Rabaan, Kuldeep Dhama, Shin Jie Yong, Firzan Nainu, Khalid Hajissa, Nourah Al Dossary, Khulood Khaled Alajmi, Afaf E. Al Saggar, Fahad Abdullah AlHarbi, Mohammed Buhays Aswany, Abdullah Abdulaziz Alshayee, Saad Abdalaziz Alrabiah, Ahmed Mahmoud Saleh, Mohammed Ali Alqarni, Fahad Mohammed Al Gharib, Shahd Nabeel Qattan, Hassan M. Almusabeh, Hussain Yousef AlGhatm, Sameer Ahmed Almoraihel, Ahmed Saeed Alzuwaid, Mohammed Ali Albaqshi, Murtadha Ahmed Al Khalaf, Yasmine Ahmed Albaqshi, Abdulsatar H Al Brahim, Mahdi Mana Al Mutared, Hassan Al-Helal, Header A Alghazal, Abbas Al Mutair

**Affiliations:** 1 Administration of Pharmaceutical Care, Al-Ahsa Health Cluster, Ministry of Health, Al-Ahsa 31982, Saudi Arabia; 2 Molecular Diagnostic Laboratory, Johns Hopkins Aramco Healthcare, Dhahran 31311, Saudi Arabia; 3 College of Medicine, Alfaisal University, Riyadh 11533, Saudi Arabia; 4 Department of Public Health/Nutrition, The University of Haripur, Haripur 22620, Khyber Pakhtunkhwa, Pakistan; 5 Division of Pathology, ICAR-Indian Veterinary Research Institute, Izatangar, Bareilly 243122, Uttar Pradesh, India; 6 Department of Biological Sciences, School of Medical and Life Sciences, Sunway University, Subang Jaya 47500, Malaysia; 7 Department of Pharmacy, Faculty of Pharmacy, Hasanuddin University, Makassar 90245, Indonesia; 8 Department of Medical Microbiology & Parasitology, School of Medical Sciences, Universiti Sains Malaysia, Kubang Kerian 16150, Malaysia; 9 General Surgery Department, Alomran General Hospital, Ministry of Health, Al-Ahsa 36358, Saudi Arabia; 10 Pharmacy Department, Al-Ahsa Mental Health Hospital, Al-Ahsa 31982, Saudi Arabia; 11 Administration of Accreditation, Quality and Performance Excellence Management, Riyadh 3rd Health Cluster, Ministry of Health, Riyadh 13717, Saudi Arabia; 12 Clinical Pharmacy Services—Nephrology Department, King Saud Hospital, Ministry of Health, Riyadh 56437, Saudi Arabia; 13 Administration of Supply and Shared Services, C1 Riyadh Health Cluster, Ministry of Health, Riyadh 11622, Saudi Arabia; 14 Administration of Compliance, Directorate of Health Affairs, Ministry of Health, Eastern Region, Al-Ahsa 36441, Saudi Arabia; 15 Diagnostic Radiology Department, Prince Sultan Military Medical City, Riyadh 12233, Saudi Arabia; 16 Clinical Pharmacy Department, Gurayat General Hospital, Ministry of Health, Gurayat 77471, Saudi Arabia; 17 Pharmacy Department, Aljafr General Hospital, Ministry of Health, Al-Ahsa 77110, Saudi Arabia; 18 Respiratory Therapy Department, Maternity and Children Hospital, Ministry of Health, Al-Ahsa 36422, Saudi Arabia; 19 Pharmacy Department, King Fahad Hofuf Hospital, Ministry of Health, Al-Ahsa 36441, Saudi Arabia; 20 Psychological Service Department, Ardha and Mental Health Complex, Ministry of Health, Najran 66248, Saudi Arabia; 21 Division of Laboratory, Medical Microbiology Department, Maternity and Children Hospital, Ministry of Health, Al-Ahsa 36422, Saudi Arabia; 22 Microbiology Laboratory, Prince Saud Bin Jalawi Hospital, Ministry of Health, Al-Ahsa 36424, Saudi Arabia; 23 Research Center, Almoosa Specialist Hospital, Al-Ahsa 36342, Saudi Arabia; 24 College of Nursing, Princess Norah Bint Abdulrahman University, Riyadh 11564, Saudi Arabia; 25 School of Nursing, Wollongong University, Wollongong, NSW 2522, Australia; 26 Department of Nursing, Prince Sultan Military College, Dharan 34313, Saudi Arabia

**Keywords:** allograft, COVID-19, disease, infection, meta-analysis, organ, rejection, SARS-CoV-2, systematic review, transplant, vaccine, vaccination

## Abstract

Background: Solid organ rejection post-SARS-CoV-2 vaccination or COVID-19 infection is extremely rare but can occur. T-cell recognition of antigen is the primary and central event that leads to the cascade of events that result in rejection of a transplanted organ. Objectives: To describe the results of a systematic review for solid organ rejections following SARS-CoV-2 vaccination or COVID-19 infection. Methods: For this systematic review and meta-analysis, we searched Proquest, Medline, Embase, Pubmed, CINAHL, Wiley online library, Scopus and Nature through the Preferred Reporting Items for Systematic Reviews and Meta Analyses (PRISMA) guidelines for studies on the incidence of solid organ rejection post-SARS-CoV-2 vaccination or COVID-19 infection, published from 1 December 2019 to 31 May 2022, with English language restriction. Results: One hundred thirty-six cases from fifty-two articles were included in the qualitative synthesis of this systematic review (56 solid organs rejected post-SARS-CoV-2 vaccination and 40 solid organs rejected following COVID-19 infection). Cornea rejection (44 cases) was the most frequent organ observed post-SARS-CoV-2 vaccination and following COVID-19 infection, followed by kidney rejection (36 cases), liver rejection (12 cases), lung rejection (2 cases), heart rejection (1 case) and pancreas rejection (1 case). The median or mean patient age ranged from 23 to 94 years across the studies. The majority of the patients were male (*n* = 51, 53.1%) and were of White (Caucasian) (*n* = 51, 53.7%) and Hispanic (*n* = 15, 15.8%) ethnicity. A total of fifty-six solid organ rejections were reported post-SARS-CoV-2 vaccination [Pfizer-BioNTech (*n* = 31), Moderna (*n* = 14), Oxford Uni-AstraZeneca (*n* = 10) and Sinovac-CoronaVac (*n* = 1)]. The median time from SARS-CoV-2 vaccination to organ rejection was 13.5 h (IQR, 3.2–17.2), while the median time from COVID-19 infection to organ rejection was 14 h (IQR, 5–21). Most patients were easily treated without any serious complications, recovered and did not require long-term allograft rejection therapy [graft success (*n* = 70, 85.4%), graft failure (*n* = 12, 14.6%), survived (*n* = 90, 95.7%) and died (*n* = 4, 4.3%)]. Conclusion: The reported evidence of solid organ rejections post-SARS-CoV-2 vaccination or COIVD-19 infection should not discourage vaccination against this worldwide pandemic. The number of reported cases is relatively small in relation to the hundreds of millions of vaccinations that have occurred, and the protective benefits offered by SARS-CoV-2 vaccination far outweigh the risks.

## 1. Introduction

Owing to the increased risk of complications associated with the severe acute respiratory syndrome coronavirus 2 (SARS-CoV-2) infection, transplant recipients are a high-risk group recommended for coronavirus disease 2019 (COVID-19) vaccination. Vaccination against SARS-CoVS-2 is considered to be the best medical solution to end the current COVID-19 pandemic, and all SARS-CoV-2 vaccines have been determined to be safe. Maintenance of vaccine safety requires a proactive approach to maintain public confidence and reduce vaccine hesitancy [1,2]. The most commonly reported side effects of SARS-CoV-2 vaccines are fever, headache, fatigue and pain at the injection site, and overall, most side effects were mild-to-moderate and self-limited [3]. COVID-19 has now been demonstrated to be a multisystem disease with complex interactions with coexisting medical conditions and causing indirect effects through immune dysregulation [4].

**Figure 1:**
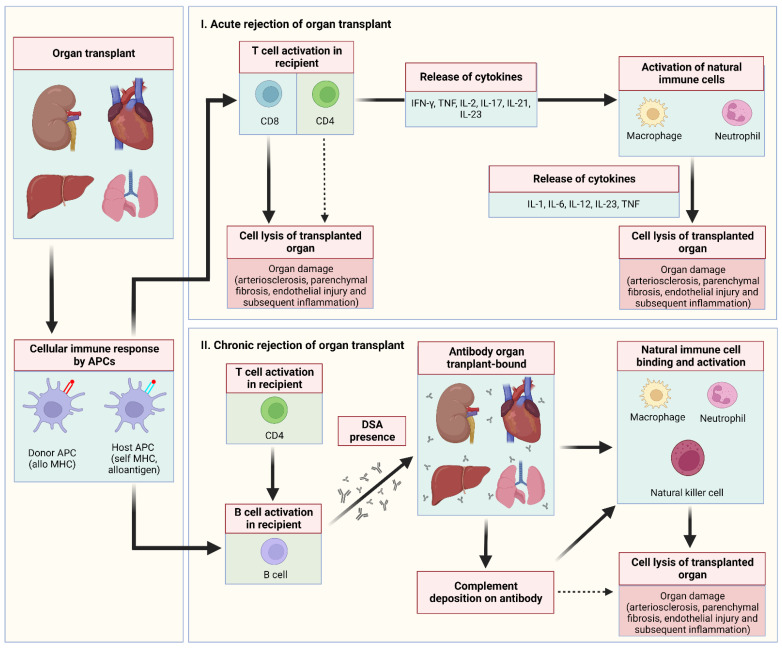
Schematic representation of intracellular signalling in solid organ rejection. In general, once T cell activation occurs, a chain of intracellular events is triggered under the influence of growth and differentiation factors. In acute rejection of organ transplant, recipient CD8 T cells and, to a lesser extent, CD4 T cells directly destroy the organ transplant. Moreover, CD4 cells in the recipient cause organ damage via the secretion of extraordinary array of cytokines with a bewildering number of functions that activate the host’s natural immune system (macrophages and neutrophils). In chronic rejection of organ transplant, donor-specific antibodies are released that bind to the organ transplant to instigate the host’s natural immune system (macrophages, neutrophils and natural killer cells) and cause complement deposition. Abbreviations: APCs, antigen-presenting cells; DSA, donor-specific antibodies; IL-1, interleukin-1; IL-2, interleukin-2; IL-6, interleukin-6; IL-12, interleukin-12; IL-17, interleukin-17; IL-21, interleukin-21; IL-23, interleukin-23; IFN-γ, interferon gamma; MHC, major histocompatibility complex; TNF, tumour necrosis factor.

Organ rejection post-COVID-19 vaccination with all vaccines used to prevent COVID-19 or following COVID-19 infection with all variants of concerns is rare but can occur. Solid organ transplant recipients may be at increased risk for COVID-19 because they are immunosuppressed and are less likely to mount effective immune responses to vaccination [5,6]. T-cell recognition of antigens is the primary and central event that leads to the cascade of events that result in rejection of a transplanted organ following SARS-CoV-2 vaccination or COVID-19 infection (see Figure 1).

A growing body of evidence has indicated that allograft rejections have occurred as a potential consequence of COVID-19 vaccines in cornea, liver and kidney transplant recipients [7,8,9,10,11]. Several cases of organ rejections following COVID-19 infection have been described among corneal and renal transplant recipients [12,13,14,15,16]. In light of newer case reports and case-series studies that were published to describe the occurrence of organ rejection following COVID-19 vaccination or post-COVID-19 infection, we provide a systematic review of the current literature to delineate the range of organ rejections that were elicited following COVID-19 vaccination or SARS-CoV-2 infection.

## 2. Methods

### 2.1. Design

We followed the Preferred Reporting Items for Systematic Reviews and Meta-Analyses guidelines (PRISMA) in conducting this systematic review and meta-analysis [17]. The following electronic databases were searched: PROQUEST, MEDLINE, EMBASE, PUBMED, CINAHL, WILEY ONLINE LIBRARY, SCOPUS and NATURE with Full Text.

We used the following keywords: *COVID-19* OR *SARS-CoV-2* OR *Severe acute Respiratory Syndrome Coronavirus 2* OR *Coronavirus Disease 2019* OR *2019 novel coronavirus* MINUS or PLUS *vaccine* OR *vaccination* AND *organ rejection* OR *transplant rejection* OR *solid organ rejection* OR *graft rejection* OR *allograft rejection* OR *cornea rejection* OR *liver transplant rejection* OR *kidney transplant rejection* OR *heart transplant rejection* OR *lung transplant rejection* OR *trachea transplant rejection* OR *pancreas transplant rejection* OR *pancreas rejection* OR *skin rejection* OR *vascular tissue rejection* OR *intestine rejection* OR *stomach rejection* OR *bowel rejection* OR *bone marrow rejection* OR *blood vessels rejection* OR *heart valve rejection* OR *bone rejection* OR *uterus rejection* OR *testis rejection* OR *penis rejection* OR *ovary rejection* OR *hand (arm) rejection* OR *shoulder rejection* OR *bladder rejection* OR *face rejection*. The search was limited to papers published in English between 1 December 2019 and 31 May 2022. Based on the title and abstract of each selected article, we selected those discussing and reporting occurrence of organ rejections due to SARS-CoV-2 vaccination or COVID-19 infection.

### 2.2. Inclusion–Exclusion Criteria

The inclusion criteria are as follows: (1) published case reports, case series and cohort studies that focused on organ rejection following SARS-CoV-2 vaccination or COVID-19 infection that included children and adults as population of interest; (2) studies of experimental or observational design reporting the incidence of organ rejection in patients post-SARS-CoV-2 vaccination or infection; and (3) the language was restricted to English.

The exclusion criteria are as follows: (1) editorials, commentaries, case and animal studies, discussion papers, preprints, news analyses, reviews and meta-analyses; (2) studies that did not report data on organ rejection due to SARS-CoV-2 vaccination or infection; (3) studies that did not report details on identified organ rejection cases following COVID-19 vaccination or infection; (4) studies that reported organ rejection in patients with no history of COVID-19 vaccination or negative SARS-CoV-2 PCR tests; and (5) duplicate publications.

### 2.3. Data Extraction

Seven authors (Saad Alhumaid, Ali A. Rabaan, Kuldeep Dhama, Shin Jie Yong, Firzan Nainu, Khalid Hajissa and Nourah Al Dossary) critically reviewed all of the studies retrieved and selected those judged to be the most relevant. Data were carefully extracted from the relevant research studies independently. Articles were categorized as case report, case series or cohort studies.

The following data were extracted from selected studies: authors; publication year; study location; study design and setting; age; proportion of male patients; patient ethnicity; time from COVID-19 vaccination to organ rejection; vaccine brand and dose (if first dose, second dose or third dose); if organ rejection is new-onset or relapsed; method used to detect COVID-19; symptoms of COVID-19 infection; time from COVID-19 infection to organ rejection; medical comorbidities; patient clinical presentation; abnormal laboratory indicators; biopsy examination and radiological imaging findings; treatment given after organ rejection; assessment of study risk of bias; if patient suffered graft failure; and final treatment outcome (survived or died).

### 2.4. Quality Assessment

The quality assessment of the studies was undertaken based on the Newcastle–Ottawa Scale (NOS) to assess the quality of the selected studies [18]. This assessment scale has two different tools for evaluating case-control and cohort studies. Each tool measures quality in the three parameters of selection, comparability and exposure/outcome and allocates a maximum of four, two and three points, respectively [18]. High-quality studies are scored greater than 7 on this scale, and moderate-quality studies scored between 5 and 7 [18]. Quality assessment was performed by six authors (Khulood Khaled Alajmi, Afaf E. Al Saggar, Fahad Abdullah AlHarbi, Mohammed Buhays Aswany, Abdullah Abdulaziz Alshayee and Saad Abdalaziz Alrabiah) independently, with any disagreement resolved by consensus.

### 2.5. Data Analysis

**Figure 2:**
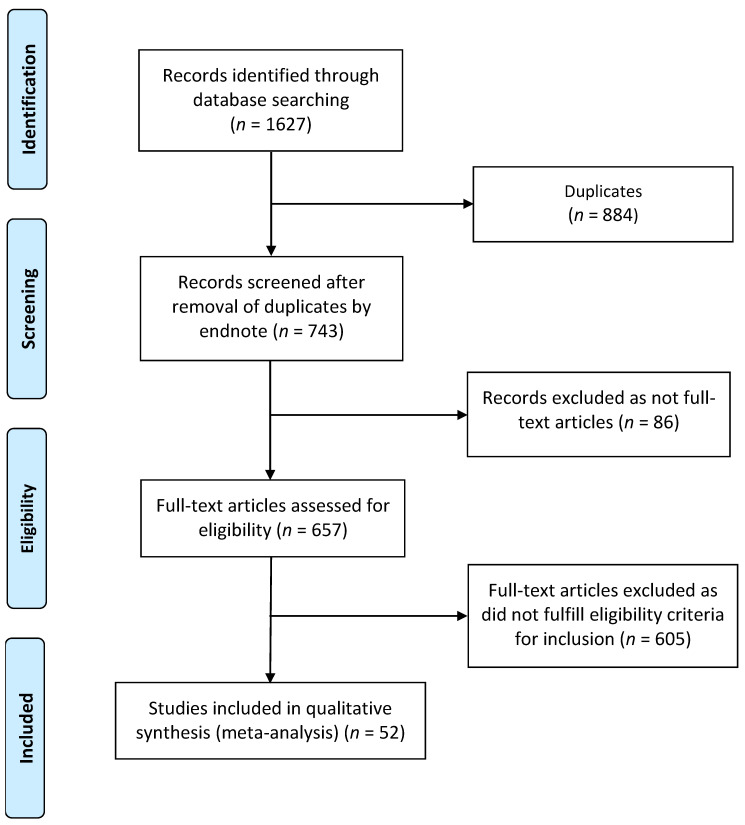
Flow diagram of literature search and data extraction from studies included in the systematic review and meta-analysis.

**Figure 3:**
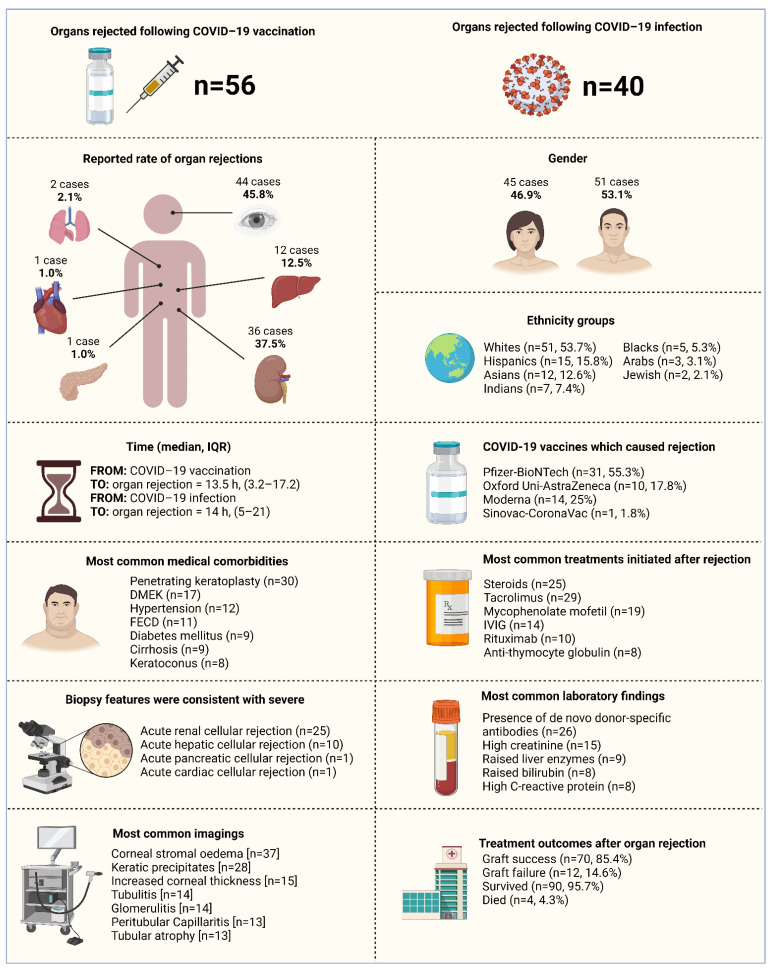
Summary of the characteristics of the included studies with evidence on organ rejection following COVID-19 vaccination and post-COVID-19 infection (*n* = 52 studies), 2020–2022. Abbreviations: COVID-19, coronavirus disease 2019; IVIG, intravenous immunoglobulin; DMEK, Descemet’s membrane endothelial keratoplasty; FECD, Fuchs endothelial corneal dystrophy.

We primarily examined the proportion of confirmed cases that suffered organ rejection due to SARS-CoV-2 vaccination or COVID-19 infection. This proportion was further classified based on the type of organ rejection induced by the SARS-CoV-2 vaccine or COVID-19 infection (i.e., if cornea, kidney, liver, heart, lung or pancreas rejection). Descriptive statistics were used to describe the data. For continuous variables, the mean and standard deviation were used to summarize the data, and for categorical variables, frequencies and percentages were reported. Microsoft Excel 2019 (Microsoft Corp., Redmond, DC, USA) was used for all statistical analyses. Figure 2 was created with Microsoft Word 2019 (Microsoft Corp., Redmond, DC, USA). Figure 1 and Figure 3 were created with BioRender.com (agreement no. IU23TYL40X) (accessed on 19 July 2022).

## 3. Results

### 3.1. Study Characteristics and Quality

A total of 1627 publications were identified (Figure 2). After the exclusion of duplicates and articles that did not fulfill the study inclusion criteria, fifty-two articles were included in the qualitative synthesis of this systematic review. The reports of ninety-six cases (fifty-six organ rejection cases following COVID-19 vaccination [7,8,9,10,11,19,20,21,22,23,24,25,26,27,28,29,30,31,32,33,34,35,36,37,38,39,40,41,42,43,44,45] and forty organ rejection cases after COVID-19 infection [12,13,14,15,16,46,47,48,49,50,51,52,53,54,55,56,57,58,59,60]) identified from these articles are presented in groups based on confirmed diagnoses, laboratory, biopsy and imaging findings. The detailed characteristics of the included studies are shown in Table 1 and Table 2. Among these, one article was in preprint version [24].

There were 42 case reports [5,6,7,8,11,12,13,17,18,20,21,22,24,25,26,27,29,30,31,32,33,34,35,37,38,39,40,41,42,43,44,45,48,49,50,51,52,53,54,55,56,57], 8 case series [9,10,14,28,36,46,47,58] and 2 cohort studies [19,23]. These studies were conducted in the United States (*n* = 14), India (*n* = 7), Italy (*n* = 5), Canada (*n* = 4), United Kingdom (*n* = 3), France (*n* = 3), Brazil (*n* = 2), Lebanon (*n* = 2), Australia (*n* = 1), Greece (*n* = 1), Egypt (*n* = 1), Denmark (*n* = 1), Japan (*n* = 1), Israel (*n* = 1), South Korea (*n* = 1), Slovakia (*n* = 1), Croatia (*n* = 1), China (*n* = 1), Mexico (*n* = 1) and Sweden (*n* = 1). The majority of the studies were single centre [5,6,7,8,9,11,12,13,17,18,20,21,22,24,25,26,27,28,29,30,31,32,33,34,35,36,37,38,39,40,41,42,43,44,45,48,49,50,51,52,53,54,55,56,57,58], and only six studies were multi-centre [10,14,19,23,46,47]. The median NOS score for these studies was 6 (range, 5–7). Among the 52 included studies, 37 studies were moderate-quality studies (i.e., NOS scores were between 5 and 7), and 15 studies demonstrated relatively high quality (i.e., NOS scores > 7); Table 1 and Table 2.

### 3.2. Meta-Analysis of Organs Rejection Following COVID-19 Vaccination

There were reports of fifty-six organ rejection cases following COVID-19 vaccination (fifty-one new-onset cases [5,6,7,8,9,17,18,19,20,21,22,23,24,25,26,27,28,29,30,31,32,33,34,35,36,37,39,40,41,42,43] and five relapsed cases [5,9,36,38]) (see Table 1). Allograft rejections after COVID-19 vaccination occurred for cornea (*n* = 38, 67.8%) [5,8,17,20,21,22,23,24,28,29,30,31,32,33,34,35,37,38,42,43], liver (*n* = 11, 19.6%) [9,25,36,39,41], kidney (*n* = 6, 10.7%) [6,7,18,19,26,40] and pancreas (*n* = 1, 1.8%) [27] transplant recipients.

The most common clinical presentations in these transplant patients who presented with organ rejection post-COVID-19 vaccination were diminished vision (*n* = 22) [5,8,17,20,22,23,24,31,33,34,35,37], eye redness (*n* = 15) [21,22,23,29,30,32,34,38,42], blurred vision (*n* = 14) [5,28,29,30,32,38,42,43], ocular pain (*n* = 14) [8,17,22,23,29,32,34,38,43], photophobia (*n* = 6) [17,32,37,38,43], weakness (*n* = 5) [25,27,34,40], myalgia (*n* = 3) [29,32,34,38] and fatigue (*n* = 3) [6,40,41].

The median interquartile range (IQR) age of this group was 63.5 (51 to 72.7) years, with a similar gender rate in patients who presented with organ rejections found after COVID-19 vaccination (female (*n* = 29) [5,7,8,9,17,21,23,26,27,28,29,30,32,34,36,37,38,39,40,41] and male (*n* = 27) [5,6,18,20,22,23,24,25,28,31,33,35,36,37,42,43]), and the majority of the patients were White (Caucasian) (*n* = 36, 64.3%) [5,6,7,8,9,17,18,21,24,25,28,30,32,34,36,37,39,40,41,43] and Asian (*n* = 9, 16.1%) [23,26] ethnicity. The median (IQR) time from COVID-19 vaccination to organ rejection was 13.5 (3.2 to 17.2) days.

Thirty-one of these fifty-six cases (seventeen after the first dose [8,9,18,19,20,23,25,28,30,32,34,36,39,42] and twelve after the second dose [7,17,23,24,26,32,36]) were reported following Pfizer-BioNTech vaccination. The remaining organ rejections cases were reported after Moderna (*n* = 14) [5,6,21,22,36,37,41,43], Oxford Uni-AstraZeneca (*n* = 10) [5,27,28,29,31,33,35,40] and Sinovac-CoronaVac (*n* = 1) [38] COVID-19 vaccination.

Thirty-seven of those patients had a medical history of eye diseases (penetrating keratoplasty (*n* = 27) [5,22,23,24,28,30,31,33,35,37,38,42,43], Descemet’s membrane endothelial keratoplasty (*n* = 16) [8,17,20,23,28,32,33,34,37], Fuchs’ endothelial corneal dystrophy (*n* = 8) [5,8,28,32,34,37], infectious keratitis (*n* = 5) [5,37,38], cataract operation (*n* = 5) [8,32,37,38], pseudophakic bullous keratopathy (*n* = 4) [5,22,33,37] and glaucoma (*n* = 2) [38,43]).

A considerable number of those patients had a medical history related to the liver (cirrhosis (*n* = 8) [9,25,36,39,41], liver transplant recipients (*n* = 8) [25,36,39,41], biliary atresia (*n* = 1) [9], hepatitis C virus (*n* = 1) [41] and hepatocellular carcinoma (*n* = 1) [41]) or kidney (end-stage kidney disease (*n* = 2) [6,40], kidney transplant recipient (*n* = 1) [6], polycystic kidney disease (*n* = 1) [9] and diabetic kidney disease (*n* = 1) [40]).

In one patient, the medical history was not reported [19], and only one patient had no medical history [21]; however, few of those reported cases had pre-existing diabetes mellitus (*n* = 5) [5,25,27,37,40] or hypertension (*n* = 5) [6,8,20,26,40]. Few of those cases presented with a previous known history of organ rejections for cornea (*n* = 2) [5,42] and liver (*n* = 2) [36].

Laboratory indices were not performed for a high number of cases who presented with organ rejection post-COVID-19 vaccination, particularly ones who suffered cornea rejections (*n* = 22, 39.3%) [5,8,17,20,21,22,23,24,28,29,31,32,33,34,35,37,38,42,43]; however, patients were more likely to have raised liver enzymes (*n* = 12) [9,25,36,39,41], raised bilirubin (*n* = 8) [9,25,36], the presence of de novo donor-specific antibodies (*n* = 5) [7,18,39,40], high creatinine (*n* = 5) [6,7,19,26,40], high C-reactive protein (*n* = 2) [6,25], thrombocytopenia (*n* = 2) [25,39] and low haemoglobin (*n* = 2) [39,40].

Biopsy for patients who presented with liver, kidney and pancreas rejections post-COVID-19 vaccination shown histopathological features consistent with acute hepatic cellular rejection (*n* = 4, 7.1%) [9,25,36,41], acute renal cellular rejection (*n* = 4, 7.1%) [6,7,26,40] and acute pancreatic cellular rejection (*n* = 1, 1.8%) [27], respectively. Most of the radiological imaging shown corneal stromal oedema (*n* = 34) [5,8,20,22,23,28,29,30,31,32,33,34,35,37,38,42,43], keratic precipitates (*n* = 24) [5,22,23,28,30,31,32,34,37,42,43], increased corneal thickness (*n* = 13) [5,8,23,38], Descemet’s membrane folds (*n* = 9) [17,22,28,29,30,34,37,42], cells in the anterior chamber (*n* = 7) [5,23,30,34,37,42], conjunctival injection (*n* = 5) [32,34,37], anterior chamber inflammation (*n* = 4) [32,34,35] and Khodadoust’s rejection line (*n* = 4) [29,35,37].

As expected, most prescribed pharmacotherapy agents in these organ rejection cases were steroids (*n* = 58) [5,6,7,8,9,17,20,21,22,23,24,25,26,27,28,29,30,31,32,33,34,36,37,38,39,40,41,42,43], tacrolimus (*n* = 11) [9,19,22,23,24,36,38], mycophenolate mofetil (*n* = 4) [9,21,36,41], IVIG (*n* = 4) [6,25,39,40] and anti-thymocyte globulin (*n* = 3) [6,27,41]. Graft failure due to organ rejection post-COVID-19 vaccination was reported in cornea (*n* = 5, 10%) [8,22,23,38,43], liver (*n* = 1, 1.8%) [9] and kidney (*n* = 1, 1.8%) [40] transplant recipients. Clinical outcomes of the organ rejection patients post-COVID-19 vaccination with mortality were documented in one (1.8%) [9], while 54 (96.4%) of the organ rejection cases recovered [5,6,7,8,9,17,18,20,21,22,23,24,25,26,27,28,29,30,31,32,33,34,35,36,37,38,39,40,41,42,43], and final treatment outcome was not reported in one case only (*n* = 1, 1.8%) [19].

### 3.3. Meta-Analysis of Organs Rejection after COVID-19 Infection

There were reports of forty organ rejection cases following COVID-19 infection [12,13,14,15,16,46,47,48,49,50,51,52,53,54,55,56,57,58,59,60] (see Table 2). Allograft rejections after COVID-19 infection occurred in kidney (*n* = 30, 75%) [12,13,16,46,47,48,49,53,55,57,60], cornea (*n* = 6, 15%) [14,15,50,56,59], lung (*n* = 2, 5%) [52,58], liver (*n* = 1, 2.5%) [54] and heart (*n* = 1, 2.5%) [51] transplant recipients. The most common clinical presentations in these transplant patients who presented with organ rejection post-COVID-19 infection were acute kidney injury (*n* = 14, 35%) [12,16,47,49,55,60], peripheral oedema (*n* = 6, 15%) [12,46,47,49], worsened vision (*n* = 6, 15%) [14,15,50,56,59], eye redness (*n* = 4, 10%) [14,15,56,59], reduced urine output (*n* = 2, 5%) [47,53], respiratory failure (*n* = 2, 5%) [52,58], eye discomfort (*n* = 2, 5%) [56,59] and conjunctivitis (*n* = 2, 5%) [56]; nevertheless, clinical presentations due to organ rejections were not reported in some patients (*n* = 5, 12.5%) [13,48,54].

The median interquartile range (IQR) age of this group was 51 (33 to 57) years, with an increased male predominance in patients who presented with organ rejections found after COVID-19 infection (*n* = 24, 60%) [12,13,16,46,47,48,49,52,53,57,58,59,60], and majority of the patients were of White (Caucasian) (*n* = 15, 37.5%) [13,14,48,49,51,52,54,55,56,58] and Hispanic (*n* = 14, 35%) [60] ethnicity. The laboratory technique of rt-PCR was used to detect SARS-CoV-2 in all patients included in this group [12,13,14,15,16,46,47,49,50,51,52,53,54,55,56,57,59,60], except for one case where the detection method of SARS-CoV-2 was not reported [58].

The most prevalent COVID-19 symptoms in these patients were fever (*n* = 16) [12,14,15,46,47,48,49,52,54,57,58,59], nausea (*n* = 5) [12,46,50], diarrhea (*n* = 5) [46,47,48,49,53], cough (*n* = 5) [12,48,49,50,52], vomiting (*n* = 4) [12,46,50,53], dyspnoea (*n* = 4) [48,49,52] and anosmia (*n* = 3) [12,14,46]. Few patients were asymptomatic for COVID-19 (*n* = 4) [13,16,49]. The median (IQR) time from COVID-19 infection to organ rejection was 14 (5 to 21) days.

Thirty of those patients had a medical history related to the kidney (end-stage kidney disease (*n* = 2) [16,46], focal and segmental glomerulosclerosis (*n* = 2) [12,57], IgA nephropathy (*n* = 2) [16,53], simultaneous pancreas and kidney transplant (*n* = 2) [13], minimal change disease (*n* = 1) [55], congenital single kidney (*n* = 1) [55], nephrosclerosis (*n* = 1) [46], lupus nephropathy (*n* = 1) [49], chronic kidney disease (*n* = 1) [51], dominant polycystic kidney disease (*n* = 1) [49] and unknown primary kidney disease (*n* = 1) [49]).

Six of those patients had a medical history of eye diseases (penetrating keratoplasty (*n* = 3) [15,50,59], Descemet’s membrane endothelial keratoplasty (*n* = 3) [14,56], Fuchs’ endothelial corneal dystrophy (*n* = 3) [14,56], glaucoma (*n* = 2) [56,59], age-related macular degeneration (*n* = 2) [56], keratoconus (*n* = 1) [15] and cataract operation (*n* = 1) [59]). Some of those reported cases had pre-existing hypertension (*n* = 7) [12,16,46,49,60], diabetes mellitus (*n* = 5) [12,46,49,51,52] and ischemic heart disease (*n* = 3) [47,51]. Few of those cases presented with a previous known history of organ rejections for kidney (*n* = 5) [55,60].

Laboratory indices were not performed for a high number of cases who presented with organ rejection post-COVID-19 infection particularly in ones who suffered kidney and cornea rejections (*n* = 19, 47.5%) [14,15,48,56,60]; however, patients were more likely to have the presence of de novo donor-specific antibodies (*n* = 21) [12,13,46,51,52,54,57,58], high creatinine (*n* = 10) [12,13,16,46,47,53,55,57], high C-reactive protein (*n* = 6) [46,47,49,53,57,59], proteinuria (*n* = 5) [12,46,49,53], high D-dimer (*n* = 3) [47,49,55] and the isolation of infectious pathogens (*n* = 3) (namely *Pseudomonas aeruginosa* and *Mycobacterium kansasii* [61] (*n* = 1) [58], *E. Faecium* (urine) (*n* = 1) [55] and *Candida* species (cornea) (*n* = 1) [50]).

Almost all biopsy examinations in patients who presented with kidney rejections post-COVID-19 infection showed histopathological features consistent with acute renal cellular rejection (*n* = 23, 57.5%) [12,46,47,53,55,57,60]; however, biopsy evaluation was not performed for many patients who were diagnosed with organ rejection due to COVID-19 infection (*n* = 10, 25%) [13,14,15,48,50,56,58,59]. Most of the radiological abnormal images were seen in patients with kidney rejection (tubulitis (*n* = 14) [60], glomerulitis (*n* = 14) [60], inflammation in non-scarred cortex (*n* = 13) [60], peritubular capillaritis (*n* = 13) [60], tubular atrophy (*n* = 13) [60] and chronic glomerulopathy (*n* = 4) [60]) and cornea rejection (keratic precipitates (*n* = 4) [14,15,50,59] and corneal stromal oedema (*n* = 4) [15,56,59]) following COVID-19 infection.

As expected, most prescribed pharmacotherapy agents in these organ rejection cases were steroid (*n* = 30) [12,13,14,15,16,47,48,50,51,52,53,55,56,57,58,59,60], tacrolimus (*n* = 18) [47,48,51,52,53,57,58,60], mycophenolate mofetil (*n* = 15) [46,51,53,57,60], IVIG (*n* = 10) [12,13,16,46,49,52,53,54,57], rituximab (*n* = 8) [12,13,52,60], antibiotics (*n* = 6) [46,47,49,50,55,58], anticoagulation (*n* = 6) [47,49,50,54], anti-thymocyte globulin (*n* = 5) [13,53,60] and haemodialysis (*n* = 5) [12,13,47,49]. Graft failure due to organ rejection post-COVID-19 infection was reported in cornea (*n* = 2, 5%) [50,56], lung (*n* = 2, 5%) [52,58] and kidney (*n* = 1, 2.5%) [47] transplant recipients. The clinical outcomes of the organ rejection patients post-COVID-19 infection with mortality were documented in three cases (7.5%) [47,52,58], while 37 (92.5%) of the organ rejection cases recovered [12,13,14,15,16,46,48,49,50,51,53,54,55,56,57,59,60].

A summary of the overall characteristics of the fifty-two studies that we included in this review with evidence on organ rejection after both COVID-19 vaccination and COVID-19 infection can be seen in Figure 3.

## 4. Discussion

A considerable number of solid organ rejections were observed following SARS-CoV-2 vaccination or COVID-19 infection. As the dominant organ rejection type following SARS-CoV-2 vaccination reported in our review, cornea allograft failure post-SARS-CoV-2 vaccines and COVID-19 infection has been increasingly well-documented in the literature during the preceding year penetrating keratoplasty or Descemet’s membrane endothelial keratoplasty [7,10,15,56]. However, cornea transplantation is the oldest, most common and arguably the most successful form of solid tissue transplantation in the human body [62].

Corneal allograft rejection occurs due to a highly complex sequence of immune responses that promote tissue destruction and major histocompatibility complex class II complex antigens in all layers of the grafted cornea are induced due to SARS-CoV-2 vaccines [7,37], which can explain the susceptibility of different organ graft types, such as kidney, liver, heart and pancreas, etc. regardless of grafting technique. The antigens presented in the anterior chamber generate noncomplement antibodies, and the formation of cytotoxic T lymphocyte precursors against the graft and the inflammatory cytokines may enhance the major histocompatibility complex expression [63].

Corneal allograft rejection has also been reported following other kinds of vaccines, such as *influenza* [64], *hepatitis B* [65], *tetanus* [65], *herpes zoster* [66] and *yellow fever* [67]. SARS-CoV-2 vaccination-associated corneal graft rejection is a rare but likely underreported phenomenon [68]. The recent and ongoing administration of billions of SARS-CoV-2 vaccine doses has brought vaccine-related corneal graft rejection into the light for healthcare workers globally [69].

Acute corneal transplant rejection had already been reported in association with COVID-19 disease [14,15,50,56]. SARS-CoV-2 has been known to infect cells via angiotensin-converting enzyme 2 receptors for entry and transmembrane serine protease 2 [70], which have been found to be expressed in human corneal epithelium [71,72]. Uncontrolled and elevated release of pro-inflammatory cytokines and suppressed immunity [73], leading to the cytokine storm triggered by COVID-19, can overcome corneal immune privilege, thus, giving rise to allograft rejection episodes.

On a higher scale, the same pathway may lead to the inflammatory immune response triggered by vaccination. There are currently no guidelines regarding either the use of SARS-CoV-2 vaccines or for the increase of anti-rejection prophylaxis before or after vaccination or post-COVID-19 infection in patients with tissue corneal allografts [22]. However, health practitioners should be alert, and patients need to be educated to follow up immediately if they notice any changes, such as diminished or altered or blurred vision, eye redness or discomfort [14,15,19,23].

If diagnosed early, corneal transplant rejection can be reversed, although there may be endothelial cell loss [74]. Based on the published case reports, the incidence of graft rejection episodes seems to peak at about 2 weeks, and increased use of topical steroids around the time of receiving a vaccine or post-keratoplasty in recipients who develop COVID-19 is advisable [19,23,56,59]. Treatment of graft rejection following SARS-CoV-2 vaccination or COVID-19 with topical and occasionally systemic corticosteroids is largely successful, similar to other types of rejection [68]. Corneal graft recipients should be encouraged to receive the SARS-CoV-2 vaccine, particularly considering the association of COVID-19 infection itself with acute corneal graft rejection.

Kidney as a target of SARS-CoV-2 can be supported by the findings of isolated virus from the urine of infected patients [75] and the fact that angiotensin-converting enzyme 2 receptors is plentifully present in renal tissue, mostly in podocytes and in the brush border of the proximal tubule [76]. While the risks of SARS-CoV-2 vaccines and COVID-19 infection in respect to the release of anti-HLA antibodies are still unclear, it is documented that some vaccines (including seasonal *influenza* and *pneumococcal* vaccines [77,78]) and infections (such as *Pseudomonas aeruginosa* [46,79]) can be associated with re-activating memory B cells leading to the presence of anti-HLA antibody production that may cause antibody-mediated rejection in kidney-transplant recipients [80].

Based on a small case-series study of patients with end-stage renal failure awaiting a kidney transplant, there was no development of anti-HLA antibodies as a result from COVID-19 infection [81]. The authors concluded that there may not be a need to repeat HLA antibody testing or perform a physical crossmatch on admission serum before kidney transplant for patients who recovered from COVID-19 [81].

When infected with COVID-19, renal allograft population displays a high risk of mortality with numbers reaching 30% to 32% compared to the 1% to 5% mortality in the general population [82,83], a negative finding, which encouraged healthcare providers to adjust the baseline immunosuppression regimen when their transplant patients become COVID-19-infected. Consequently, an allograft renal rejective effect is most likely because of reducing the dose of immunosuppressive drugs taken by patients to help overcome COVID-19 infection [84,85].

To add insult to injury, direct kidney infection, disturbance of the renin-angiotensin-aldosterone homeostasis and the pro-inflammatory cytokine milieu may contribute to the subsequent renal complications [86]. A balanced regimen of the immunosuppressants and prescribing appropriate dosages to allow proper immune response to the invading SARS-CoV-2 while keeping transplanted kidney allografts tolerable to recipient’s immune system is considered a challenge in the era of COVID-19 [87]. The severity of COVID-19 could potentially be affected by the type, combinations and intensity of immunosuppression.

For instance, lymphocyte-depleting antibodies or antimetabolites cause lymphopenia, which is a reported risk factor for severe COVID-19 illness [88]. Mycophenolate may impair the ability to develop an adequate immune response to natural infection resulting in lower immunogenicity [89,90]. Therefore, antimetabolites (e.g., mycophenolate mofetil) are recommended to be held or reduced in particular for patients with lymphopenia (absolute lymphocyte count of less than 700 cells/mL) and calcineurin inhibitors (e.g., tacrolimus and cyclosporine A) should generally be continued as they inhibit interleukin-6 and interleukin-1 pathways [5,91].

Despite the previously documented effects of other vaccines and COVID-19 infection on antibodies formation, with no previous history of allergy, no COVID-19 infection and no autoimmunity, should be considered as a potential limitation of SARS-CoV-2 vaccination for patients on renal transplant waiting lists [92]. By comparison, the risk of COVID-19-related morbidity and mortality is much greater compared with the risk of vaccination-related kidney allograft rejection [8]. It is worth considering monitoring graft function after vaccination against SARS-CoV-2 by examination of serum creatinine, proteinuria and de novo donor-specific antibodies.

Although there is much less concern that SARS-CoV-2 vaccines and COVID-19 infection could lead to immunologically mediated rejection of the liver [27,38,41,54], heart [51] or pancreas [29], luckily, the acceptance rate for COVID-19 vaccination among recipients with these types of organ transplants is extremely high [93,94,95,96].

Suspicion for a potentially causal association between SARS-CoV-2 vaccination or COVID-19 infection and development of liver, heart or pancreas cellular rejection may be raised due to the timing of allograft rejection onset and the presence of typical risk factors with organ rejection (old age, preformed or de novo DSA, prior organ rejection, inadequate immunosuppression adherence or drug levels and autoimmune organ disease aetiology) [97,98,99]. It is important to note that all cases of acute cellular rejection of the liver, heart and pancreas post-SARS-CoV-2 vaccination or COVID-19 infection included in this review were easily treated without any serious complications except for one patient with liver allograft who contracted COVID-19 during a workup for retransplantation and died from its complications [11].

As the humoral immune response to SARS-CoV-2 vaccines is impaired in solid organ transplant recipients compared to the general population [100,101,102], a third dose is approved by the American Food and Drug Administration and the Centres for Disease Control and Prevention and highly recommended [103,104] and evidence for a fourth dose has only recently been established [105,106] in this special group of patients and shown to improve the immune response without causing short-term or serious adverse events. So, this highlights the need of close monitoring of the allograft population when a transplant recipient plans to undergo COVID-19 vaccination.

Although the immunogenicity and efficacy of COVID-19 vaccines are lower in solid organ transplant recipients than the general population [100,101,102], the benefit from vaccination outweighs risk for most patients. Vaccination is recommended to be delayed for at least one month from the time of transplantation and for at least three months after use of T cell-depleting agents (e.g., anti-thymocyte globulin) or specific B cell-depletion agents (e.g., rituximab) [61]. Another strategy to provide protection in receipts of solid organ transplants and taking transplant-related immunosuppressive drugs is the use of anti-SARS-CoV-2 monoclonal antibodies.

The monoclonal antibody combination tixagevimab-cilgavimab is a potential option for pre-exposure prophylaxis against COVID-19 for solid organ transplant individuals who may not benefit maximally from vaccination and for those who have a contraindication to vaccination [107]. Solid organ transplant recipients who have had close contact with an individual with SARS-CoV-2 infection or who are at high risk of exposure to individuals with infection in an institutional setting are eligible for prophylactic monoclonal antibody treatment.

Due to their immunosuppressed state, all exposed solid organ transplant recipients for COVID-19 are typically referred to post-exposure prophylaxis using the monoclonal antibody combinations casirivimab-imdevimab [108] or bamlanivimab-etesevimab [109] to prevent SARS-CoV-2 infection. However, the availability of those monoclonal antibodies is limited, and it should be noted that pre-exposure and post-exposure prophylaxis is not a substitute for vaccination. Last but not least, artificial intelligence has been shown to be an emerging and promising technology for detecting early COVID-19 infection and monitoring the state of affected individuals [110] as well as a powerful tool for low-cost, fast and large-scale SARS-CoV-2 vaccine effectiveness evaluation [111].

### Limitations

First, while most of the evidence discussed was based on limited case series and many case reports, many of these were small and performed in single centres and not necessarily generalizable to the current COVID-19 vaccination settings or patients infected with SARS-CoV-2. Second, all studies included in this review were retrospective in design, which could have introduced potential reporting bias due to reliance on clinical case records. Third, the study population included adult patients, and hence its results cannot be generalized to paediatric patients.

## 5. Conclusions

A range of solid organ rejections post-SARS-CoV-2 vaccination or following COVID-19 infection may occur at an extremely rare rate and is likely to be immune-mediated. Reported evidence of allograft rejection post-SARS-CoV-2 vaccination or following COIVD-19 infection should not discourage vaccinating this most vulnerable human subpopulation. The number of reported cases is relatively small in relation to the hundreds of millions of vaccinations that have occurred, and the protective benefits offered by SARS-CoV-2 vaccination far outweigh the risks.

## Figures and Tables

**Table 1:** Summary of the characteristics of the included studies with evidence on organ rejection post-COVID-19 vaccination (*n* = 32 studies), 2021–2022.

Author, Year, Study Location	Study Design, Setting	Age (Years) ^a^	Male, *N* (%)	Ethnicity ^b^	Time from COVID-19 Vaccination to Organ Rejection (Days)	Comorbidities, *N*	Vaccine Brand and Dose	New Onset or Relapse	Clinical Presentation	Laboratory Findings	Biopsy Findings ^c^	Imaging	Treatment Initiated after Rejection, *N*	NOS Score; Graft Failure; and Treatment Outcome
**Organ rejected: LIVER**														
Hughes et al. 2022 [27], United States	Retrospective case report, single centre	65	1 [100]	1 White (Caucasian)	2	1 Cryptogenic cirrhosis 1 Liver transplant recipient 1 Coronary artery disease 1 Diabetes mellitus 1 Hyperlipidaemia	Pfizer-BioNTech, dose 1 [*n* = 1]	New-onset [*n* = 1]	1 Extremity weakness 1 Paraesthesia ascending to bilateral hands 1 Hyporeflexia 1 Loss of pinprick sensation 1 Difficulty with walking 1 Bilateral cranial nerve 7 palsies 1 Acute inflammatory demyelinating polyneuropathy	1 Raised liver enzymes 1 Raised bilirubin 1 Thrombocytopenia 1 Raised white blood cells 1 High C-reactive protein	Mild AHCR in patient’s graft [*n* = 1]	Innumerable new bilobar lesions [*n* = 1]	1 IVIG 1 Steroid	(NOS, 7) No [*n* = 1] 1 survived
Hume et al. 2022 [11], Australia	Retrospective case-series, single centre	30.7 ± 15.1	0 [0]	3 Whites (Caucasians)	Mean [SD], 11.3 [3]	1 Cryptogenic cirrhosis 1 Caroli’s disease 1 Autosomal recessive polycystic kidney disease 1 Biliary atresia	Pfizer-BioNTech, dose 1 [*n* = 3]	New-onset [*n* = 2] Relapsed [*n* = 1]	1 Liver allograft failure 1 Positive PCR for SARS-CoV-2	3 Raised liver enzymes 3 Raised bilirubin	Moderate or severe AHCR in patient’s graft [*n* = 1]	Not reported [*n* = 3]	3 Steroid 3 Tacrolimus 1 Mycophenolate mofetil 1 Ursodeosxycholic acid 1 Plasma exchange 1 Rituximab	(NOS, 8) No [*n* = 2] Yes [*n* = 1] 2 survived 1 died
Sarwar et al. 2022 [38], United States	Retrospective case-series, single centre	54 (51–66)	4 [80]	5 Whites (Caucasians)	Mean [SD], 11.6 [4.6]	5 Liver transplant recipients 3 Non-alcoholic steatohepatitis-related cirrhosis 2 Alcohol-related cirrhosis 2 History of acute cellular rejection	Moderna, dose 1 and dose 2 [*n* = 3] Pfizer-BioNTech, dose 1 and dose 2 [*n* = 2]	New-onset [*n* = 3] Relapsed [*n* = 2]	Not reported [*n* = 5]	3 Raised liver enzymes 4 Raised bilirubin	Typical features of T cell-mediated AHCR including portal inflammation of predominantly mixed activated lymphocytes, portal vein phlebitis and bile duct injuries [*n* = 5]	Not performed [*n* = 5]	9 Steroid 1 Everolimus 2 Tacrolimus 1 Cyclosporine 1 Mycophenolate mofetil	(NOS, 6) No [*n* = 5] 5 survived
Valsecchi et al. 2022 [41], Italy	Retrospective case report, single centre	58	0 [0]	1 White (Caucasian)	44	1 Autoimmune cirrhosis 1 Grade II encephalopathy 1 Refractory ascites 1 End-stage liver disease 1 Liver transplant recipient	Pfizer-BioNTech, dose 1 [*n* = 1]	New-onset [*n* = 1]	1 Worsened neurologic status 1 Vaccine-induced immune thrombotic thrombocytopenia 1 Graft-versus-host disorder 1 Transplantation-mediated alloimmune thrombocytopenia	1 Low haemoglobin 1 Thrombocytopenia 1 High INR 1 High D-dimer 1 Raised liver enzymes 1 Positive for antibodies directed against (PF4) antibodies	Not performed [*n* = 1]	Small millimetric high density area on the occipital lobe [*n* = 1]	1 Heparin 1 Fondaparinux 1 IVIG 1 Steroid	(NOS, 7) No [*n* = 1] 1 survived
Vyhmeister et al. 2021 [43], United States	Retrospective case report, single centre	64	0 [0]	1 White (Caucasian)	11	1 Cirrhosis 1 Hepatitis C virus 1 Hepatocellular carcinoma 1 Liver transplant recipient	Moderna, dose 1 [*n* = 1]	New-onset [*n* = 1]	1 Dark urine 1 Fatigue 1 Malaise	1 Raised liver enzymes	Typical features of AHCR including mixed portal inflammation, bile duct injury and endotheliitis [*n* = 1]	Unremarkable [*n* = 1]	1 Steroid 1 Azathioprine 1 Mycophenolate mofetil 1 Anti-thymocyte globulin	(NOS, 6) No [*n* = 1] 1 survived
**Organ rejected: CORNEA**														
Abousy et al. 2021 [19], United States	Retrospective case report, single centre	73	0 [0]	1 White (Caucasian)	14	1 Bilateral Descemet stripping endothelial keratoplasty	Pfizer-BioNTech, dose 2 [*n* = 1]	New-onset [*n* = 1]	1 Bilateral decreased visual acuity 1 Ocular pain 1 Photophobia	Not performed [*n* = 1]	Not performed [*n* = 1]	Quiet conjunctiva and sclera [*n* = 1] Bilateral thickened corneas with Descemet folds [*n* = 1]	1 Steroid 1 Sodium chloride hypertonicity	(NOS, 7) No [*n* = 1] 1 survived
Balidis et al. 2021 [7], Greece	Retrospective case reports, single centre	66.5 (63.2–75)	2 [50]	4 Whites (Caucasians)	7 (5.5–9.2)	1 Pseudophakic bullous keratopathy 4 Penetrating keratoplasty 1 Fuch’s endothelial corneal dystrophy 1 Hyperdense nuclear cataract 1 Graft rejection on 3 different occasions 1 Herpes simplex keratitis 1 Diabetes mellitus 1 Diabetic macular oedema 1 Herpetic keratitis 1 Extensive post-herpetic corneal scarring	Moderna, dose 1 [*n* = 1] and dose 2 [*n* = 1] Oxford Uni-AstraZeneca, dose 1 [*n* = 2]	New-onset [*n* = 3] Relapsed [*n* = 1]	2 Blurred vision 2 Gradual deterioration of vision	Not performed [*n* = 4]	Not performed [*n* = 4]	Subtle corneal oedema [*n* = 4] Small pigmented keratic precipitates [*n* = 4] Subepithelial bullae 1 Cells ( + ) in the anterior chamber [*n* = 1] Increased corneal thickness [*n* = 3]	4 Steroid 2 Hypertonic eye drops	(NOS, 8) No [*n* = 4] 4 survived
Crnej et al. 2021 [22], Lebanon	Retrospective case report, single centre	71	1 [100]	1 Arab	7	1 Hypertension 1 Smoking 1 Coronary artery disease 1 Descemet’s membrane endothelial keratoplasty	Pfizer-BioNTech, dose 1 [*n* = 1]	New-onset [*n* = 1]	1 Painless decrease of vision	Not performed [*n* = 1]	Not performed [*n* = 1]	Diffuse corneal oedema [*n* = 1]	1 Steroid 1 Valacyclovir	(NOS, 6) No [*n* = 1] 1 survived
de la Presa et al. 2022 [23], United States	Retrospective case report, single centre	27	0 [0]	1 White (Caucasian)	15	1 No medical history	Moderna, dose 1 [*n* = 1]	New-onset [*n* = 1]	1 Acute redness and irritation of the right eye	Not performed [*n* = 1]	Not performed [*n* = 1]	1+ conjunctival hyperemia [*n* = 1] Irregular epithelial rejection line [*n* = 1] Epitheliopathy [*n* = 1]	1 Steroid 1 Difluprednate 1 Mycophenolate mofetil	(NOS, 7) No [*n* = 1] 1 survived
Eleiwa et al. 2022 [24], Egypt	Retrospective case report, single centre	81	1 [100]	1 Arab	3	1 Penetrating keratoplasty 1 Pseudophakic bullous keratopathy	Moderna, dose 2 [*n* = 1]	New-onset [*n* = 1]	1 Painful pink eye 1 Rapid decline in vision 1 Mild flu-like illness	Not performed [*n* = 1]	Not performed [*n* = 1]	Diffuse corneal punctate staining [*n* = 1] Diffuse severe corneal graft oedema [*n* = 1] Descemet’s folds [*n* = 1] Scattered keratic precipitates [*n* = 1]	1 Steroid 1 Tacrolimus 1 Acyclovir 1 Bandage contact lens was inserted	(NOS, 5) Yes [*n* = 1] 1 survived
Forshaw et al. 2022 [10], Denmark	Retrospective case report, single centre	94	0 [0]	1 White (Caucasian)	14	1 Fuchs’ endothelial dystrophy 1 Bilateral Descemet membrane endothelial keratoplasty 1 Hypertension 1 Cataract operation	Pfizer-BioNTech, dose 1 [*n* = 1]	New-onset [*n* = 1]	1 Rapid decline in vision 1 Ocular pain	Not performed [*n* = 1]	Not performed [*n* = 1]	Diffuse corneal oedema [*n* = 1] Increased corneal thickness [*n* = 1]	1 Steroid 1 Antibiotics 1 Sodium chloride hypertonicity 1 Analgesics 1 re-Descemet membrane endothelial keratoplasty transplantation	(NOS, 8) Yes [*n* = 1] 1 survived
Fujimoto et al. 2021 [25], Japan	Retrospective cohort, multicentre	80 (50–87)	5 [71.4]	7 Asians	Mean [SD], 69 [35.8]	7 Penetrating keratoplasty 3 Descemet stripping automated endothelial keratoplasty 2 Anterior lamellar keratoplasty 2 Corneal limbal transplantation	Pfizer-BioNTech, dose 1 [*n* = 1] Pfizer-BioNTech, dose 2 [*n* = 6]	New-onset [*n* = 7]	7 Painful pink eye 7 Rapid decline in vision	Not performed [*n* = 1]	Not performed [*n* = 1]	Bullous keratopathy [*n* = 1] Corneal stromal oedema [*n* = 7] Cells in the anterior chamber [*n* = 1] Keratic precipitates [*n* = 7] Increased corneal thickness [*n* = 7]	6 Steroid 2 Tacrolimus 1 Acyclovir	(NOS, 7) No [*n* = 6] Yes [*n* = 1] 7 survived
Gouvea et al. 2022 [26], Canada	Retrospective case report, single centre	72	1 [100]	1 White (Caucasian)	30	1 Total limbal stem cell deficiency 1 Penetrating keratoplasty	Pfizer-BioNTech, dose 2 [*n* = 1]	New-onset [*n* = 1]	1 Rapid decline in vision	Not performed [*n* = 1]	Not performed [*n* = 1]	Circumferential perilimbal engorgement [*n* = 1] Stagnation [*n* = 1] Tortuosity of vessels with mild chemosis [*n* = 1]	1 Difluprednate 1 Tacrolimus	(NOS, 6) No [*n* = 1] 1 survived
Molero-Senosiain et al. 2022 [30], United Kingdom	Retrospective case-series, single centre	61 (51.5–77)	2 [40]	4 Whites (Caucasians) 1 Asian	Mean [SD], 16.86 [6.96] for Pfizer-BioNTech Mean [SD], 17 [11.89] for Oxford Uni-AstraZeneca	2 Descemet stripping automated endothelial keratoplasty 2 Fuchs endothelial dystrophy 3 Penetrating keratoplasty 3 Keratoconus	Pfizer-BioNTech, dose 1 [*n* = 3] Oxford Uni-AstraZeneca, dose 2 [*n* = 2]	New-onset [*n* = 5]	5 Blurred vision	Not performed [*n* = 1]	Not performed [*n* = 1]	Diffuse corneal graft oedema [*n* = 5] Descemet folds [*n* = 2] Localized keratic precipitates [*n* = 1] Mild anterior chamber reaction [*n* = 1]	5 Steroid	(NOS, 8) No [*n* = 5] 5 survived
Nahata et al. 2022 [31], India	Retrospective case report, single centre	28	0 [0]	1 Indian	14	1 Pellucid marginal degeneration 1 Femtosecond laser enabled keratoplasty	Oxford Uni-AstraZeneca, dose 1 [*n* = 1]	New-onset [*n* = 1]	1 Ocular pain 1 Eye redness 1 Blurring of vision	Not performed [*n* = 1]	Not performed [*n* = 1]	Stromal oedema with Descemet’s membrane folds [*n* = 1] Khodadoust line with anterior chamber cells [*n* = 1] Flare [*n* = 1]	1 Steroid 1 Cycloplegics	(NOS, 6) No [*n* = 1] 1 survived
Nioi et al. 2021 [32], Italy	Retrospective case report, single centre	44	0 [0]	1 White (Caucasian)	13	1 Penetrating keratoplasty 1 Keratoconus	Pfizer-BioNTech, dose 1 [*n* = 1]	New-onset [*n* = 1]	1 Blurred vision 1 Eye redness 1 Eye discomfort	1 Vitamin D deficiency	Not performed [*n* = 1]	Ciliary injection [*n* = 1] Diffuse corneal oedema within the graft [*n* = 1] Keratic precipitates [*n* = 1] Descemet folds [*n* = 1] Anterior chamber cells [*n* = 1]	1 Steroid 1 Vitamin D supplement	(NOS, 8) No [*n* = 1] 1 survived
Parmar et al. 2021 [33], India	Retrospective case report, single centre	35	1 [100]	1 Indian	2	1 Penetrating keratoplasty	Oxford Uni-AstraZeneca, dose 1 [*n* = 1]	New-onset [*n* = 1]	1 Diminished vision	Not performed [*n* = 1]	Not performed [*n* = 1]	Microcystic epithelial and stromal corneal graft oedema [*n* = 1] Few fresh endothelial keratic precipitates [*n* = 1]	1 Steroid 1 Cycloplegics	(NOS, 6) No [*n* = 1] 1 survived
Phylactou et al. 2021 [34], United Kingdom	Retrospective case reports, single centre	66 and 83	0 [0]	2 Whites (Caucasians)	7 and 21	1 Human immunodeficiency virus infection 2 Fuchs endothelial corneal dystrophy 2 Descemet’s membrane endothelial keratoplasty 1 Cataract operation	Pfizer-BioNTech, dose 1 [*n* = 1] Pfizer-BioNTech, dose 2 [*n* = 1]	New-onset [*n* = 2]	2 Blurred vision 2 Eye redness 2 Photophobia 1 Ocular pain	Not performed [*n* = 1]	Not performed [*n* = 1]	Moderate conjunctival injection [*n* = 2] Diffuse corneal oedema [*n* = 1] Fine keratic precipitates [*n* = 2] Anterior chamber inflammation [*n* = 2]	2 Steroid	(NOS, 8) No [*n* = 2] 2 survived
Rajagopal et al. 2022 [35], India	Retrospective case report, single centre	79	1 [100]	1 Indian	42	1 Penetrating keratoplasty 1 Removed right eye 1 Endophthalmitis 1 Descemet’s stripping endothelial keratoplasty 1 Pseudophakic bullous keratopathy 1 Hodgkin’s lymphoma	Oxford Uni-AstraZeneca, dose 2 [*n* = 1]	New-onset [*n* = 1]	1 Diminished vision	Not performed [*n* = 1]	Not performed [*n* = 1]	Central stromal oedema [*n* = 1]	1 Steroid	(NOS, 6) No [*n* = 1] 1 survived
Rallis et al. 2021 [36], United Kingdom	Retrospective case report, single centre	68	0 [0]	1 White (Caucasian)	4	1 Bilateral lamellar Descemet Stripping Automated Endothelial Keratoplasty 1 Fuchs’ corneal endothelial dystrophy 1 Left re-do penetrating keratoplasty	Pfizer-BioNTech, dose 1 [*n* = 1]	New-onset [*n* = 1]	1 Painful red eye 1 Rapid deterioration of vision 1 Moderate systemic reactions 1 Chills 1 Myalgia 1 Tiredness	Not performed [*n* = 1]	Not performed [*n* = 1]	Conjunctival injection [*n* = 1] Corneal graft haze [*n* = 1] Diffuse corneal oedema [*n* = 1] Descemet’s folds [*n* = 1] Scattered keratic precipitates [*n* = 1] Anterior chamber inflammation [*n* = 1] 1+ cells in anterior chamber [*n* = 1]	1 Steroid 1 Acyclovir	(NOS, 8) No [*n* = 1] 1 survived
Ravichandran et al. 2021 [37], India	Retrospective case report, single centre	62	1 [[100]]	1 Indian	21	1 Penetrating keratoplasty	Oxford Uni-AstraZeneca, dose 1 [*n* = 1]	New-onset [*n* = 1]	1 Congestion and diminished vision	Not performed [*n* = 1]	Not performed [*n* = 1]	Khodadoust’s rejection line [*n* = 1] Corneal oedema [*n* = 1] Anterior chamber inflammation [*n* = 1]	1 Not reported [*n* = 1]	(NOS, 6) No [*n* = 1] 1 survived
Shah et al. 2022 [39], United States	Retrospective case reports, single centre	71.5 (63–76.2)	2 [50]	3 Whites (Caucasians) 1 Black	14 (10.2–19.2)	2 Descemet’s membrane endothelial keratoplasty 1 Pseudophakic bullous keratopathy 1 Contact lens–related Aspergillus keratitis 1 Tectonic sclerokeratoplasty 2 Penetrating keratoplasty 2 Cataract operation 1 Chamber intraocular lens placement 1 Accidental blunt trauma (limited keratoplasty wound dehiscence) 1 Type 2 diabetes mellitus 1 Nonprogressive Salzmann nodular degeneration (left eye) 1 Fuchs endothelial corneal dystrophy 1 Multiple sclerosis	Moderna, dose 1 [*n* = 1] Moderna, dose 2 [*n* = 3]	New-onset [*n* = 4]	4 Decreased vision in the operated eye 1 Photophobia 1 Brow ache	Not performed [*n* = 1]	Not performed [*n* = 1]	Khodadoust’s rejection line [*n* = 2] Microcystic epithelial and stromal oedema [*n* = 4] Descemet membrane folds [*n* = 1] Keratic precipitates [*n* = 3] Conjunctival injection [*n* = 2] Anterior chamber cells [*n* = 1]	3 Steroid 1 Difluprednate	(NOS, 8) No [*n* = 4] 4 survived
Simão et al. 2022 [40], Brazil	Retrospective case report, single centre	63	0 [0]	1 Hispanic	1	1 Penetrating keratoplasty 1 Laser in situ keratomileusis 1 *Acanthamoeba* keratitis 1 Radial keratotomy 1 Fungal keratitis 1 Cataract operation 1 Intraocular lens implantation 1 Trabeculectomy with mitomycin-C 1 Pupilloplasty 1 Glaucoma 1 History of vaccination included influenza vaccine	Sinovac-CoronaVac, dose 1 [*n* = 1]	Relapsed [*n* = 1]	1 Blurred vision 1 Ocular pain 1 Photophobia 1 Eye redness 1 Myalgia	Not performed [*n* = 1]	Not performed [*n* = 1]	Corneal oedema [*n* = 1] Interface fluid accumulation [*n* = 1] Ciliary injection [*n* = 1] Increased corneal thickness [*n* = 1] Anterior chamber reaction [*n* = 1]	1 Steroid 1 Polydimethylsiloxane 1 Tacrolimus 1 Timolol 1 Bimatoprost	(NOS, 6) Yes [*n* = 1] 1 survived
Wasser et al. 2021 [44], Israel	Retrospective case reports, single centre	73 and 56	2 [100]	2 Jewish	13 and 14	2 Penetrating keratoplasty 1 Keratoconus 1 Regraft due to late endothelial failure 1 Keratoconus	Pfizer-BioNTech, dose 1 [*n* = 2]	New-onset [*n* = 2]	1 Eye discomfort 1 Blurred vision 1 Eye redness	Not performed [*n* = 1]	Not performed [*n* = 1]	Ciliary injection [*n* = 1] Corneal oedema [*n* = 2] Descemet folds [*n* = 1] Keratic precipitates [*n* = 2] Anterior chamber cells [*n* = 1]	2 Steroid	(NOS, 6) No [*n* = 2] 2 survived
Yu et al. 2022 [45], United States	Retrospective case report, single centre	51	1 [100]	1 White (Caucasian)	3	1 Keratoconus 1 Penetrating keratoplasty 1 Radial keratotomy 1 Glaucoma	Moderna, dose 1 [*n* = 1]	New-onset [*n* = 1]	1 Eye pain 1 Photophobia 1 Blurred vision	Not performed [*n* = 1]	Not performed [*n* = 1]	Corneal oedema [*n* = 1] Endothelial keratic precipitates [*n* = 1]	1 Steroid	(NOS, 7) Yes [*n* = 1] 1 survived
**Organ rejected: KIDNEY**														
Abu-Khader et al. 2022 [20], Canada	Retrospective case report, single centre	42	1 [100]	1 White (Caucasian)	18	1 Renal transplant waitlist 1 History of vaccination included influenza, pneumococcal conjugate; and pneumococcal polysaccharide 23 vaccines	Pfizer-BioNTech, dose 1 [*n* = 1]	New-onset [*n* = 1]	1 No clinical presentation	1 Presence of de novo donor-specific antibodies and strongly positive T and B cells	Not performed [*n* = 1]	Not performed [*n* = 1]	1 Transplant team cancelled the surgery	(NOS, 6) No [*n* = 1] 1 survived
Al Jurdi et al. 2022 [21], United States	Prospective cohort, multicentre	Not reported [*n* = 1]	Not reported [*n* = 1]	Not reported [*n* = 1]	40	Not reported [*n* = 1]	Pfizer-BioNTech, dose 1 [*n* = 1]	New-onset [*n* = 1]	Not reported [*n* = 1]	1 High creatinine 1 High urinary CXCL9 mRNA	Not reported [*n* = 1]	Not reported [*n* = 1]	1 Tacrolimus 1 Belatacept	(NOS, 6) 1 outcome was not reported
Bau et al. 2022 [8], Canada	Retrospective case report, single centre	53	1 [100]	1 White (Caucasian)	1	1 Hypertension 1 Obstructive sleep apnea 1 Obesity 1 End-stage kidney disease 1 Preemptive living-related kidney transplant	Moderna, dose 2 [*n* = 1]	New-onset [*n* = 1]	1 Fatigue 1 Muscle aches 1 Low blood pressure 1 Acute tubular injury 1 Minimal tubular atrophy	1 High creatinine 1 New mild proteinuria	Histopathological features were consistent with severe T-cell mediated ARCR [*n* = 1]	Unremarkable [*n* = 1]	1 IV fluids 1 Steroid 1 Antithymocyte globulin 1 IVIG 1 Plasmapheresis	(NOS, 8) No [*n* = 1] 1 survived
Del Bello et al. 2021 [9], France	Retrospective case report, single centre	23	0 [0]	1 White (Caucasian)	8	1 Nephronophthisis	Pfizer-BioNTech, dose 2 [*n* = 1]	New-onset [*n* = 1]	1 Impaired kidney function	1 High creatinine 1 Presence of de novo donor-specific antibodies	Histopathological features were consistent with ARCR [*n* = 1]	Not performed [*n* = 1]	1 Steroid 1 Polyclonal antibodies	(NOS, 8) No [*n* = 1] 1 survived
Jang et al. 2021 [28], South Korea	Retrospective case report, single centre	78	0 [0]	1 Asian	15	1 Hypertension 1 Herpes zoster infection	Pfizer-BioNTech, dose 2 [*n* = 1]	New-onset [*n* = 1]	1 Headache 1 Fever	1 High creatinine	Histopathological features were consistent with ARCR [*n* = 1]	Swelling of the transplanted kidney [*n* = 1]	1 Steroid	(NOS, 7) No [*n* = 1] 1 survived
Vnučák et al. 2022 [42], Slovakia	Retrospective case report, single centre	25	0 [0]	1 White (Caucasian)	14	1 Diabetic kidney disease 1 End-stage kidney disease 1 Type 1 diabetes mellitus 1 Hypertension 1 Autoimmune thyroiditis	Oxford Uni-AstraZeneca, dose 1 [*n* = 1]	New-onset [*n* = 1]	1 Fatigue 1 General weakness 1 Vomiting 1 Inability to eat or drink 1 High risk of septic complications	1 High creatinine 1 High urea 1 Low haemoglobin 1 High C-reactive protein 1 Low pH 1 Presence of de novo donor-specific antibodies 1 Leukocytosis 1 *Escherichia coli* (urine culture)	Histopathological features were consistent with ARCR [*n* = 1]	Unremarkable [*n* = 1]	1 Steroid 1 IV fluids 1 Immunosuppressants 1 IVIG 1 Plasmapheresis 1 Diuretics 1 Rituximab	(NOS, 7) Yes [*n* = 1] 1 survived
**Organ rejected: PANCREAS**														
Masset et al. 2021 [29], France	Retrospective case report, single centre	51	0 [0]	1 White (Caucasian)	1	1 Type 1 diabetes mellitus	Oxford Uni-AstraZeneca, dose 1 [*n* = 1]	New-onset [*n* = 1]	1 Weakness 1 Fever 1 Polyuria 1 Polydipsia 1 Hyperglycemia 1 Ketoacidosis	1 Elevation of lipasemia 1 Decline of the C-peptide level 1 Eosinophilia 1 Positive auto-antibodies for anti-ZnT8, anti-GAD65 and anti-islet cell	Histopathological features were consistent with APCR [*n* = 1]	Unremarkable [*n* = 1]	1 Steroid 1 Antithymocyte globulin	(NOS, 8) No [*n* = 1] 1 survived

Abbreviations: AHCR, acute hepatic cellular rejection; APCR, acute pancreatic cellular rejection; ARCR, acute renal cellular rejection; COVID-19, coronavirus disease 2019; IVIG, IV immunoglobulin; NOS, Newcastle Ottawa Scale; SD, standard deviation; SARS-CoV-2, severe acute respiratory syndrome coronavirus 2; IV, intravenous. ^a^ Data are presented as median (25th–75th percentiles), or mean ± [SD]. ^b^ Patients with black ethnicity include African-American, Black African, African and Afro-Caribbean patients. ^c^ Biopsy findings are reported based on each institution’s written report. Biopsies were not independently reviewed.

**Table 2:** Summary of the characteristics of the included studies with evidence on organ rejection post-COVID-19 infection (*n* = 20 studies), 2020–2022.

Author, Year, Study Location	Study Design, Setting	Age (Years) ^a^	Male, *N* (%)	Ethnicity ^b^	Method Used to Detect COVID-19	Symptoms of COVID-19 Infection	Time from COVID-19 Infection to Organ Rejection (Days)	Comorbidities, *N*	Clinical Presentation	Laboratory Findings	Biopsy Findings ^c^	Imaging	Treatment initiated after rejection, *n*	NOS score; Graft Failure; and Treatment Outcome
**Organ rejected: KIDNEY**														
Abuzeineh et al. 2021 [46], United States	Retrospective case report, single centre	45	1 [100]	1 Black	rt-PCR [*n* = 1]	1 Fever 1 Watery diarrhoea 1 Nausea 1 Vomiting 1 Loss of taste and smell 1 Increased lethargy 1 Reduced oral intake 1 Dry mucous membranes 1 Hypoxemia	73	1 Diabetes mellitus 1 End-stage kidney disease 1 Hypertensive nephrosclerosis	1 Weight gain 1 Bilateral lower limb and scrotal oedema 1 Hypertension	1 Presence of de novo donor-specific antibodies 1 Elevated plasma donor-derived cell-free deoxyribonucleic acid 1 High creatinine 1 High body urea nitrogen 1 High ferritin 1 High erythrocyte sedimentation rate 1 High C-reactive protein 1 High interleukin-6 1 Proteinuria	Histopathological features were consistent with ARCR [*n* = 1]	Bilateral coarse crepitations over lower lung zones [*n* = 1] Bilateral peripheral patchy opacities [*n* = 1] Mild hydronephrosis (renal allograft) [*n* = 1]	1 IVIG 1 Mycophenolate mofetil 1 IV fluids 1 Oxygen supplementation 1 Antibiotics 1 Antifungals 1 Acyclovir 1 Prone position 1 Tocilizumab 1 Inserted Foley’s catheter 1 Diuretics	(NOS, 7) No [*n* = 1] 1 survived
Akilesh et al. 2021 [12], United States	Retrospective case-series, multicentre	47 and 54	1 [50]	1 Black and 1 Asian	rt-PCR [*n* = 2]	1 Sore throat 1 Nasal congestion 1 Anosmia 1 Cough 1 Malaise 1 Pleuritic chest 1 Pain 2 Fever 1 Nausea 1 Vomiting 1 Acute respiratory failure	4 and 42	1 Human immunodeficiency virus infection 2 Hypertension 1 Diabetes mellitus 1 Focal segmental glomerulosclerosis	2 Acute kidney injury 1 Oedema	2 High creatinine 1 Low haemoglobin 1 Thrombocytopenia 2 Proteinuria 1 Presence of de novo donor-specific antibodies	Histopathological features were consistent with ARCR [*n* = 2]	Immunoglobulin A nephropathy [*n* = 1] Focal segmental glomerulosclerosis [*n* = 1] Thrombotic microangiopathy [*n* = 1]	2 Steroid 2 Antihypertensives 2 Diuretics 2 Haemodialysis 1 IVIG 1 Rituximab 1 Plasma exchange	(NOS, 8) No [*n* = 2] 2 survived
Anandh et al. 2021 [47], India	Retrospective case report, single centre	56	1 [100]	1 Indian	rt-PCR [*n* = 1]	1 Fever 1 Diarrhoea 1 Tachypnea 1 Low oxygen saturations	14	1 High dose of supplemental Vitamin C 1 Ischemic heart disease 1 Percutaneous transluminal coronary angioplasty	1 Reduced urine output 1 Swelling of legs 1 Progressive breathlessness 1 Acute tubular injury 1 Extensive oxalate crystal deposition 1 Deterioration of cardiac function (Ejection fraction of 20%)	1 High creatinine 1 Raised serum pro-BNP level 1 High C-reactive protein 1 High D-dimer	Histopathological features were consistent with ARCR [*n* = 1] Presence of extensive oxalate deposition in the tubules [*n* = 1]	Spherical spiked particles in the glomerular capillary endothelium [*n* = 1] Tubulo-reticular inclusions [*n* = 1] Moderate left ventricular dysfunction [*n* = 1] Mosaic attenuation of both lungs [*n* = 1] Ground glass opacities [*n* = 1]	1 IV fluids 1 Antibiotics 1 Steroid 1 HCQ 1 Zinc 1 Vitamin C 1 Tacrolimus 1 Haemodialysis 1 Anticoagulation 1 Remdesivir	(NOS, 7) Yes [*n* = 1] 1 died
Asti et al. 2021 [48], Italy	Retrospective case-series, multicentre	59 and 51	2 [100]	2 Whites (Caucasians)	IgG anti SARS-CoV-2 and SARS-CoV-2 nucleic capsid protein [*n* = 2]	2 Fever 1 Cough 2 Diarrhoea 1 Nausea 1 Phlegm 1 Asthenia 2 Dyspnoea 1 Conjunctivitis	Not reported [*n* = 2]	Not reported [*n* = 2]	Not reported [*n* = 2]	Not reported [*n* = 2]	Not reported [*n* = 2]	Not reported [*n* = 2]	1 Cyclosporine 2 Steroids 1 Tacrolimus	(NOS, 7) No [*n* = 2] 2 survived
Barros et al. 2020 [13], United States	Retrospective case reports, single centre	53 and 46	1 [50]	2 Whites (Caucasians)	rt-PCR and IgG anti SARS-CoV-2 [*n* = 2]	1 Mild COVID-19 1 Asymptomatic COVID-19	20 and not reported [*n* = 1]	2 Simultaneous pancreas and kidney transplant	Not reported [*n* = 2]	1 Elevated lipase levels 1 High creatinine 2 High HbA1c 1 Presence of de novo donor-specific antibodies	Not reported [*n* = 2]	Fat stranding surrounding both kidney and pancreas allografts [*n* = 1]	1 Steroid 1 Plasma exchange 1 Rituximab 1 IVIG 2 Anti-thymocyte globulin 1 Haemodialysis 1 Stent placement	(NOS, 8) No [*n* = 2] 2 survived
Basic-Jukic et al. 2021 [49], Croatia	Retrospective case-series, multicentre	40, 53 and 31	1 [33.3]	3 Whites (Caucasians)	rt-PCR [*n* = 3]	2 Fever 1 Cough 1 Dyspnoea 1 Diarrhoea 1 Asymptomatic COVID-19	Not reported [*n* = 3]	1 Lupus nephropathy 1 Autosomal dominant polycystic kidney disease 1 Unknown primary kidney disease 3 Arterial hypertension 1 Diabetes mellitus 1 Peripheral upper arm embolization 1 Disseminated cryptococcal infection	1 Acute tubular injury 1 Proteinuria 3 Peripheral oedema	1 High C-reactive protein 3 High leucocytes 1 High D-dimer	Inflammatory infiltration within the tubulointerstitial department [*n* = 1] Mononuclear infiltration [*n* = 1] Mild tubulitis [*n* = 1] Capillaritis [*n* = 1]	Bilateral imaging confirmed pneumonia [*n* = 3]	3 Anticoagulation 1 Antibiotics 1 Haemodialysis 2 IVIG	(NOS, 7) No [*n* = 3] 3 survived
Kudose et al. 2020 [16], United States	Retrospective case-series, multicentre	54	1 [100]	1 Balck	rt-PCR [*n* = 1]	1 Asymptomatic COVID-19	Not reported [*n* = 1]	1 End-stage kidney disease 1 IgA nephropathy 1 Hypertension 1 Obesity	1 Acute kidney injury	1 High creatinine 1 Low haemoglobin	Severe lymphocytic tubulitis [*n* = 1] Focal interstitial fibrosis [*n* = 1] Mild vascular sclerosis [*n* = 1]	Unremarkable [*n* = 1]	1 Tocilizumab 1 IVIG 1 Steroids	(NOS, 8) No [*n* = 1] 1 survived
Ma et al. 2022 [53], China	Retrospective case report, single centre	32 and 33	2 [100]	2 Asian	rt-PCR [*n* = 2]	1 Nausea 1 Vomiting 1 Diarrhoea	Not reported [*n* = 2]	1 IgA nephropathy	1 Glomerulonephritis 1 Polyuria 1 Foamy urine 1 Nocturia 1 Stomachache 1 Reduced urine output	2 High creatinine 2 Proteinuria 1 High C-reactive protein	Histopathological features were consistent with ARCR [*n* = 2]	Not reported [*n* = 2]	2 Steroids 2 Mycophenolate mofetil 2 Tacrolimus 1 IVIG 1 Antithymocyte globulin	(NOS, 6) No [*n* = 2] 2 survived
Mohamed et al. 2021 [55], United States	Retrospective case report, single centre	33	0 [0]	1 White (Caucasian)	rt-PCR and IgG anti SARS-CoV-2 [*n* = 1]	1 Shortness of breath 1 Pulse-oximetry (SpO2) ranging from 55–78% 1 Hypoxia 1 Tachypnea 1 Labored breathing 1 2 plus pitting oedema	5	1 Congenital single kidney 1 Minimal change disease 1 Non-ischemic cardiomyopathy 1 Mitral valve repair 1 Obstructive sleep apnea 1 Failed living-related kidney transplant 1 Ureteric stent	1 Acute kidney injury 1 Isolated vasculitis	1 High creatinine 1 High D-dimer 1 Hematuria 1 1 Isolation of *E. Faecium* (bacteriuria)	Histopathological features were consistent with ARCR [*n* = 1]	New diffuse airspace opacities [*n* = 1] Severe intimal arteritis and hyperplasia [*n* = 1]	1 Endotracheal intubation 1 Mechanical ventilation 1 Bilevel positive airway pressure 1 Convalescent plasma 1 Remdesivir 1 Antibiotics 1 Oxygen supplementation 1 Steroid	(NOS, 8) No [*n* = 1] 1 survived
Nourié et al. 2022 [57], Lebanon	Retrospective case report, single centre	54	1 [100]	1 Arab	rt-PCR [*n* = 1]	1 Fatigue 1 Fever	Not reported [*n* = 1]	1 Focal and segmental glomerulosclerosis 1 Haemodialysis	1 Global glomerulitis 1 Moderate capillaritis 1 Thrombotic microangiopathy affecting arterioles and glomeruli	1 High C-reactive protein 1 Raised white blood cells 1 High creatinine 1 Presence of de novo donor-specific antibodies	Histopathological features were consistent with ARCR [*n* = 1]	Multiple well-defined ground glass opacities [*n* = 1]	1 Acetaminophen 1 Oral hydration 1 Mycophenolate mofetil 1 Tacrolimus 1 Steroids 1 IVIG 1 Plasma exchange	(NOS, 6) No [*n* = 1] 1 survived
Vásquez-Jiménez et al. 2022 [60], Mexico	Retrospective case-series, single centre	34 (30–37)	10 (71.4)	14 Hispanics	rt-PCR [*n* = 14]	Not reported [*n* = 14]	Not reported [*n* = 14]	1 Hypertension 2 Retransplants 4 Previous rejections	8 Acute kidney injuries	Not reported [*n* = 14]	Histopathological features were consistent with ARCR [*n* = 14]	Tubulitis [*n* = 14] Glomerulitis [*n* = 14] Inflammation in non-scarred cortex [*n* = 13] Peritubular capillaritis [*n* = 13] Tubular atrophy [*n* = 13] Chronic glomerulopathy [*n* = 4] Endarteritis [*n* = 3]	10 Steroids 10 Mycophenolate mofetil 10 Tacrolimus 2 Azathioprine 2 Anti-thymocyte globulin 5 Rituximab	(NOS, 6) No [*n* = 14] 14 survived
**Organ rejected: LIVER**														
Merli et al. 2021 [54], Italy	Retrospective case report, single centre	50	0 [0]	1 White (Caucasian)	rt-PCR and IgG anti SARS-CoV-2 [*n* = 1]	1 Fever	Not reported [*n* = 1]	1 Sclerosing cholangitis 1 Refractory ascites 1 Tacrolimus-induced sinusoidal obstruction syndrome	Not reported [*n* = 1]	14 Presence of de novo donor-specific antibodies	Histopathological features were consistent with AHCR [*n* = 1]	Not reported [*n* = 1]	1 Anticoagulation 1 Defibrotide 1 Plasma exchange 1 Human albumin 1 IVIG 1 Velpatasvir and sofosbuvir	(NOS, 7) No [*n* = 1] 1 survived
**Organ rejected: CORNEA**														
Behera et al. 2021 [50], India	Retrospective case report, single centre	57	0 [0]	1 Indian	rt-PCR [*n* = 1]	1 Nausea 1 Vomiting 1 Cough 1 Mild breathlessness	2	1 Penetrating keratoplasty	1 Acute-onset painful diminution of vision 1 Injury with vegetative matter	1 Isolation of *Candida* species (cornea)	Not performed [*n* = 1]	Central corneal ulcer [*n* = 1] Stromal thinning [*n* = 1] Ground glass opacities [*n* = 1] Keratic precipitates [*n* = 1] Posterior synechiae [*n* = 1] Inflammatory iris nodules 3+ [*n* = 1] Anterior chamber cells [*n* = 1]	1 Antibiotics 1 Antifungals 1 Steroid 1 Cycloplegics 1 Lubricants 1 Anticoagulation 1 Oxygen supplementation	(NOS, 6) Yes [*n* = 1] 1 survived
Bitton et al. 2021 [14], France	Retrospective case report, single centre	60	0 [0]	1 White (Caucasian)	rt-PCR and IgG anti SARS-CoV-2 [*n* = 1]	1 Anosmia 1 Fever 1 Arthralgia	21	1 Fuch’s dystrophy 1 Descemet’s Membrane Endothelial Keratoplasty	1 Eye redness 1 Vision loss	Not reported [*n* = 1]	Not performed [*n* = 1]	Mild conjunctival hyperemia [*n* = 1] Multiple granulomatous keratic precipitates [*n* = 1] Deep anterior chamber with 1+ cells [*n* = 1] Increased corneal thickness [*n* = 1]	1 Steroid 1 Cyclosporine	(NOS, 6) No [*n* = 1] 1 survived
Jin et al. 2021 [15], United States	Retrospective case report, single centre	31	0 [0]	1 Black	rt-PCR and IgG anti SARS-CoV-2 [*n* = 1]	1 Dysgeusia 1 Fever	5	1 Asthma 1 Obstructive sleep apnea 1 Obesity 1 Bilateral keratoconus 1 Penetrating keratoplasty	1 Ocular pain 1 Eye redness 1 Worsened vision	Not reported [*n* = 1]	Not performed [*n* = 1]	Conjunctival injection [*n* = 1] Increased corneal thickness [*n* = 1] Microcystic and stromal oedema [*n* = 1] Diffuse keratic precipitates [*n* = 1]	1 Steroid	(NOS, 7) No [*n* = 1] 1 survived
Moriyama et al. 2022 [56], Brazil	Retrospective case report, single centre	77 and 69	0 [0]	2 Whites (Caucasians)	rt-PCR [*n* = 2]	Not reported [*n* = 1]	Not reported [*n* = 1]	2 Descemet’s membrane endothelial keratoplasty 2 Fuchs dystrophy 2 Age-related macular degeneration 1 Glaucoma	2 Conjunctivitis 1 Mild ocular discomfort 1 Tearing 1 Eye redness 2 Worsened vision 1 Mild transient inflammatory ocular symptoms	Not reported [*n* = 1]	Not performed [*n* = 1]	Mild corneal oedema [*n* = 2]	2 Steroid 1 A new Descemet membrane endothelial keratoplasty procedure	(NOS, 6) No [*n* = 1] Yes [*n* = 1] 2 survived
Singh et al. 2021 [59], India	Retrospective case report, single centre	32	1 [100]	1 Indian	rt-PCR [*n* = 1]	1 Sore throat 1 Fever 1 Malaise 1 Acute respiratory distress syndrome	21	1 Penetrating keratoplasty 1 Cataract operation 1 Posterior chamber intraocular lens implantation 1 Glaucoma	1 Diminished vision 1 Eye redness 1 Eye discomfort	1 High interleukin-6 1 High C-reactive protein 1 High lactate dehydrogenase	Not performed [*n* = 1]	Multiple epithelial bullae [*n* = 1] Diffuse stromal oedema [*n* = 1] Few descemet folds [*n* = 1] Keratic precipitates [*n* = 1]	1 Steroid	(NOS, 6) No [*n* = 1] 1 survived
**Organ rejected: HEART**														
Hanson et al. 2022 [51], Canada	Retrospective case report, single centre	57	0 [0]	1 White (Caucasian)	rt-PCR [*n* = 1]	1 Hypoxemia 1 Shortness of breath	7	1 Ischemic cardiomyopathy 1 Heart failure 1 Cardiogenic shock 1 Deterioration of cardiac function (Ejection fraction of 11%) 1 Ex-smoker 1 Atrial fibrillation 1 Diabetes mellitus 1 Chronic kidney disease 1 Transient ischemic attack 1 Chronic obstructive pulmonary disease	1 Increased oxygen requirements	1 Presence of de novo donor-specific antibodies	Histopathological features were consistent with ACCR [*n* = 1]	Pleural effusion [*n* = 1] Ground-glass lung phenotype [*n* = 1]	1 Steroid 1 Tacrolimus 1 Mycophenolate mofetil 1 Acetylsalicylic acid 1 Pravastatin	(NOS, 7) No [*n* = 1] 1 survived
**Organ rejected: LUNG**														
Lindstedt et al. 2021 [52], Sweden	Retrospective case report, single centre	62	1 [100]	1 White (Caucasian)	rt-PCR [*n* = 1]	1 Hypoxia 1 Dyspnoea 1 Cough 1 Fever 1 SARS-CoV-2-induced acute respiratory distress syndrome	Not reported [*n* = 1]	1 Diabetes mellitus 1 Myocardial infarction	1 Cerebral haemorrhage 1 Bloodstream infections 1 Respiratory failure 1 End-stage lung disease 1 Development of cor pulmonale	1 Presence of de novo donor-specific antibodies	Non-specific inflammation [*n* = 1] Scattered fibrosis deposits [*n* = 1]	Progressive lung disease [*n* = 1] Bilateral airspace opacities [*n* = 1] Diffuse consolidation [*n* = 1] Air bronchograms [*n* = 1] Ground-glass opacities [*n* = 1] Consolidation [*n* = 1] Interstitial thickening [*n* = 1]	1 Steroid 1 Plasmapheresis 1 Endotracheal intubation 1 Rituximab 1 IVIG 1 Tacrolimus 1 Remdesivir 1 Prone position 1 Extracorporeal membrane oxygenation (for 6 months) 1 Percutaneous tracheostomy 1 Dornase alfa 1 Mechanical ventilation	(NOS, 7) Yes [*n* = 1] 1 died
Palleschi et al. 2020 [58], Italy	Retrospective case report, single centre	31	1 [100]	1 White (Caucasian)	Not reported [*n* = 1]	1 Fever	Not reported [*n* = 1]	1 Cystic fibrosis	1 Bilateral bronchorrhea 1 Persistent hyperpyrexia 1 Mild respiratory failure 1 Dyspnoea	1 Presence of de novo donor-specific antibodies 1 Chronic colonization of *Pseudomonas aeruginosa* and *Mycobacterium kansasii*	Not performed [*n* = 1]	Bilateral confluent diffuse airspace opacities [*n* = 1]	1 Mechanical ventilation 1 Oxygen supplementation 1 Tacrolimus 1 Steroids 1 Azathioprine 1 Antibiotics 1 Antifungals 1 Ethambutol 1 Plasmapheresis 1 Endotracheal intubation	(NOS, 7) Yes [*n* = 1] 1 died

Abbreviations: ACCR, acute cardiac cellular rejection; AHCR, acute hepatic cellular rejection; ARCR, acute renal cellular rejection; COVID-19, coronavirus disease 2019; IVIG, IV immunoglobulin; NOS, Newcastle Ottawa Scale; rt-PCR, reverse transcription polymerase chain reaction; SD, standard deviation; SARS-CoV-2, severe acute respiratory syndrome coronavirus 2; IV, intravenous; HCQ, hydroxychloroquine; BNP, B-type natriuretic peptide. ^a^ Data are presented as median (25th–75th percentiles), or mean ± [[SD]]. ^b^ Patients with black ethnicity include African-American, Black African, African and Afro-Caribbean patients. ^c^ Biopsy findings are reported based on each institution’s written report. Biopsies were not independently reviewed.

## Data Availability

Not applicable.

## Abbreviations

ACCR	Acute Cardiac Cellular Rejection
AHCR	Acute hepatic cellular rejection
APCR	acute pancreatic cellular rejection
ARCR	acute renal cellular rejection
COVID-19	Coronavirus disease 2019
NOS	Newcastle–Ottawa scale
PRISMA	Preferred Reporting Items for systematic reviews and meta-Analyses
SARS-CoV-2	Severe acute respiratory syndrome coronavirus 2
